# Performance Evaluation of Multi-Modal Radar Signal Processing in Dense Co-Existent Environments

**DOI:** 10.3390/s26082317

**Published:** 2026-04-09

**Authors:** Anum Pirkani, Fatemeh Norouzian, Ali Bekar, Muge Bekar, Marina Gashinova

**Affiliations:** 1 School of Electronic, Electrical and Systems Engineering, University of Birmingham, Birmingham B15 2TT, UK; f.norouzian@bham.ac.uk (F.N.); a.bekar@bham.ac.uk (A.B.); muge.bekar@agu.edu.tr (M.B.); m.s.gashinova@bham.ac.uk (M.G.); 2 Department of Electrical and Electronics Engineering, Nigde Omer Halisdemir University, Nigde 51240, Turkey; 3 Department of Electrical and Electronics Engineering, Abdullah Gul University, Kayseri 38080, Turkey

**Keywords:** automotive radar, beamforming, Doppler Beam Sharpening, interference suppression, maritime radar, MIMO, MIMO-DBS, multi-modal, mutual interference, radar interference, self-interference, synthetic aperture radar

## Abstract

The wide-scale deployment of radars, distributed across a platform and across multiple platforms for reliable 360° situational awareness (SA), introduces the challenge of radar interference. Interference can broadly be categorised as self-interference (between radars mounted on the same platform) and mutual interference (signals received from radars on other platforms). Both types of interference impede the reliability of SA delivered by such systems, particularly in dense environments where numerous radars operate simultaneously within the same frequency band. This work presents a comprehensive evaluation of a multi-modal beamforming approach that combines unfocused synthetic aperture radar with the traditional Multiple-Input, Multiple-Output beamformer to enhance radar resolution and suppress interference. Additionally, various aspects of sensor configurations defining hardware and software capabilities of state-of-the-art radars are discussed, and a systematic analysis of signal-to-interference-plus-noise ratio at each step of the processing is presented. Extensive simulations and experimental results in both automotive and maritime environments are shown to validate the effectiveness of the proposed approach.

## 1. Introduction

Radar sensors are a key enabling technology for advanced automation and situational awareness (SA). In automotive applications, they support functions such as adaptive cruise control, real-time traffic monitoring, lane-change assistance, and collision avoidance. These capabilities have inspired recent progress in maritime autonomy, particularly for small- and medium-sized platforms operating in open-sea, harbour, and coastal waters [1]. In these environments, visibility is frequently degraded by spray, rain, and fog, which can severely reduce the reliability of electro-optical sensors. Radar, by contrast, remains operational under adverse conditions [2], making it well-suited for autonomy tasks, e.g., navigation and path planning, detection of flotsam/jetsam, docking, and infrastructure monitoring.

In the dense co-existent scenarios where platforms share the same space, signals from radars might interfere, causing a degradation in radar sensing and imaging performance [3]. The interfering signals could be received directly from other sensors, mutual interference, or be a result of multipath, where reflections from the infrastructure mask the echo signal or result in ghost objects [4].

Automotive environments are inherently dense, whereas in the maritime environment, compared with sparse open-sea conditions, harbour and coastal areas are cluttered and rich in reflective structures, where the probability of interference occurrence is higher. Furthermore, there are trends of installing multiple sensors across a platform for 360-degree imagery [5], which increases the probability of self-interference, where radars distributed across a single platform interfere with each other. This could either be through the antenna’s sidelobes or when the signal bounces off the nearby infrastructure and is received by another radar on the same platform. Consequently, there is a growing need for sensing methods that provide high resolution while remaining robust to such dynamics and complex propagation.

Interference detection, avoidance, and mitigation have been extensively studied for automotive radars, whereas interference in maritime environments has not yet been analysed to the best of the authors’ knowledge. In automotive environments, interference detection in the temporal domain uses techniques such as envelope detection [6]; image processing through maximally stable extremal regions to detect interference based on its distinct pattern [7]; and morphological component analysis to decompose the received signal into interference and echo return [8]. Avoidance strategies include techniques such as frequency hopping of host radar (HR) signal to minimise probability of concurrent operation of host and interfering radars in the same frequency band [9,10], chirp randomisation [11], and the use of digital waveforms such as orthogonal frequency division multiplexing to differentiate signals of different radars [12,13]. While effective in certain settings, these methods require modifications at both software and hardware levels, which limits deployability on state-of-the-art (SoA) chipsets.

Interference mitigation (IM) methods commonly exploit temporal [14], spatial [15,16], and spectral [10,17] characteristics of interference and can be broadly categorised into: (i) design-based methods using specialised waveforms such as digital coding sequences [18] and hardware-based measures and (ii) signal-processing methods that are typically integrated into the receive chain for detection and suppression of interference, e.g., by zeroing it out [19], detection and reconstruction of the useful signal, or optimisation-based approaches that differentiate between interference and useful signals. However, most of these techniques may only be effective when interfering radar has specific waveform parameters, may be ineffective in dense environments [8,15], or may lead to reduced echo signal power, particularly when dealing with multiple interference sources or when the interference source is proximal to the target [11]. Deep learning-based approaches [20,21] have also shown promise but often require extensive training data and are computationally intensive. To overcome such limitations, cognitive IM strategies, where radars adjust their waveform characteristics in response to the changing environment [6,22,23], have also been discussed, though like other methods, their performance is also compromised in dense environments where interference signals arrive from multiple directions [8,15].

Alongside interference suppression, high-resolution imagery is increasingly desirable for reliable SA, enabling scene segmentation, classification [24] and moving target detection and tracking. Contemporary radars frequently employ Multiple-Input, Multiple-Output (MIMO) arrays to improve angular resolution within a compact footprint [25]. However, MIMO arrays suffer from higher sidelobe levels (SLLs), beam widening off-boresight and performance degradation in the background of interference [11].

To achieve high-resolution imagery across the field-of-view (FoV), multi-modal beamforming, where a MIMO beamformer is combined with synthetic beamforming techniques, such as synthetic aperture radar (SAR) and Doppler Beam Sharpening (DBS)—an unfocused SAR—is essential. By combining complementary angle estimates from MIMO and SAR/DBS beamformers, prior studies have shown improved resolution and enhanced signal-to-noise ratio (SNR) [26,27] while remaining computationally efficient [1]. In our previous work, we developed the methodology for multi-modal beamforming and demonstrated its resolution benefits for improved echo signal detection in interference-free and thermal-noise-limited conditions.

The primary objective of this work is to analyse the robustness of such multi-modal beamforming in interference-limited conditions, which has not been reported before to the best of the authors’ knowledge. We specifically focus on different environments, including automotive and maritime, and quantified the MIMO-DBS interference-suppression capability across distinct interference classes in these environments. In a vast majority of cases, interference pulses are incoherent at the low pass filter (LPF) output [28]. This paper investigates how this incoherence can be exploited within a multi-modal framework for interference suppression. The main contributions of this study are:

1. We investigate the effect of MIMO-DBS under interference conditions for two cases: (a) a stationary scene, where only the platform equipped with HR is moving, and (b) a dynamic scene, where both the HR and the targets are in motion.
2. We characterise the operational boundaries of multi-modal beamforming in automotive and maritime conditions, including multi-interferer scenarios.
3. We present a quantitative analysis of signal-to-interference-plus-noise ratio (SINR) and interference-to-noise ratio (INR) at each stage of the radar processing chain, identify critical situations and parameters and assess their impact on radar functionality.
4. We validate the analysis using data collected in automotive and maritime environments.

The remainder of the paper is structured as follows: Section 2 overviews multi-modal processing and discusses its impact on interference. Section 3 presents an analytical estimate of SINR and INR across the processing chain. Section 4 presents INR heatmaps for representative interference classes and evaluates the performance of multi-modal processing in a multi-interferer scenario. Section 5 describes the experimental methodology and discusses the measured results. Section 6 presents the conclusions and future work.

## 2. Signal Processing Framework

Multi-modal processing based on MIMO and DBS in the background of thermal noise has been discussed in our previous work [1]. Here, we briefly summarise it with emphasis on: (i) analysis of the echo signal at thermal noise background (absence of interference) as a reference case and (ii) echo signal reception in the presence of interference.

The HR transmits a sequence of Frequency Modulated Continuous Wave (FMCW) chirps, $N_{CPI}$, with a chirp repetition interval, CRI, during a coherent processing interval (CPI) of duration $t_{F}=N_{CPI}\;\cdot \;CRI$. Phase variations across chirps within a Doppler frame provide an estimate of the relative velocity of targets. Spatial resolution is obtained using MIMO beamforming, where orthogonal signals are transmitted by *M* transmit (Tx) elements and, after being reflected, are received by *N* receive (Rx) elements. Let $t\in [0,T_{\mathrm{sv}}]$ denote fast-time within a chirp and $t_{s}=n\;\mathrm{CRI}$ denote slow-time, with $n=0,\ldots ,N_{\mathrm{CPI}}-1$.

### 2.1. Multi-Modal Processing for Echo Signal

First, we consider a reference scenario (no interference), where the platform equipped with HR moves with velocity $v_{p}$ and observes a stationary target. The received signal is processed by the analogue front end (AFE), followed by down-conversion, after which the phase of the intermediate frequency (IF) signal, $\phi_{\mathrm{IF}-t}\left(t,t_{s}\right)$ can be expressed as

(1) $$\phi_{\mathrm{IF}-t}\left(t,t_{s}\right)=2\pi \left(-0.5\;K_{v}\;\tau_{v}^{2}\left(t_{s}\right)+K_{v}\;t\;\tau_{v}\left(t_{s}\right)+F_{v}\;\tau_{v}\left(t_{s}\right)\right),$$

where $K_{v}$ is the HR sweep rate, $F_{v}$ is the HR carrier frequency, and $\tau_{v}\left(t_{s}\right)$ is the two-way propagation delay of the echo return. The corresponding IF frequency is obtained by differentiating (1) with respect to fast-time to obtain $f_{\mathrm{IF}-t}\left(t_{s}\right)=K_{v}\;\tau_{v}\left(t_{s}\right)$, which is constant with respect to *t*, but varies over slow-time with changing range. The digitised signal is then compressed along fast-time and slow-time to obtain range and Doppler (radial velocity) estimates, followed by digital beamforming for spatial resolution.

#### 2.1.1. Doppler Beam Sharpening: Echo Signal

**Figure 1:**
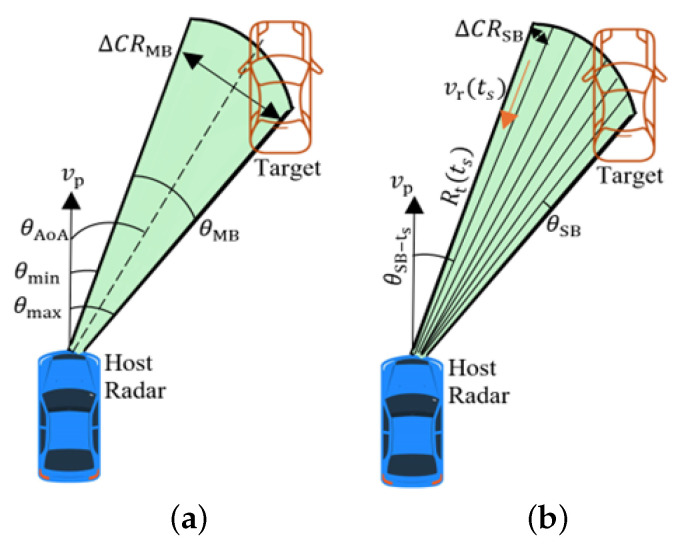
DBS principle. (**a**) Physical beam; (**b**) DBS sub-beams.

DBS leverages differences in the relative Doppler frequency shifts of scatterers at different angles relative to the trajectory of a moving platform to synthetically narrow down the physical beam, illustrated in Figure 1. As the HR moves, a scattering point traverses the physical beam, and the return signal will have an aspect angle-dependent Doppler shift. By dividing the Doppler bandwidth by Doppler resolution, the physical beam (Figure 1a) can be presented by sub-beams (Figure 1b). With fine Doppler resolution, multiple scatterers within the physical beam can be resolved to form an image.

The radial velocity across each sub-beam (Doppler bin) is $v_{r}\left(t_{s}\right)=-v_{p}\cos\theta_{SB-t_{s}}$, where $\theta_{SB-t_{s}}$ is the angle of arrival (AoA) of the signal received at slow-time $t_{s}$. For a stationary scene observed by a moving radar, the two-way Doppler is $f_{D-t}=\frac{2v_{p}}{\lambda }\cos\theta_{\mathrm{SB}-t_{s}}$. Hence, the Doppler can be mapped to the angle (DBS angle) as

(2) $$\theta_{\mathrm{SB}-t_{s}}=\arccos\;\left(f_{D-t}\;\frac{\lambda }{2v_{p}}\right).$$

#### 2.1.2. MIMO Beamforming: Echo Signal

In MIMO mode, orthogonal waveforms are transmitted such that the received signal can be associated with each Tx element [11], forming a virtual aperture of $N_{\mathrm{MIMO}}=M\;\cdot \;N$ elements. After spatial compression, the azimuth angle is estimated as

(3) $$\theta_{t\mathrm{-MIMO}}=\arcsin\;\left(\frac{\Delta \phi_{\mathrm{MIMO}}}{k\;d}\right),$$

where $\Delta \phi_{\mathrm{MIMO}}$ is the phase difference between consecutive virtual elements, k=2π/λ is the wavenumber, and d=λ/2 is the element spacing.

#### 2.1.3. MIMO-DBS Beamforming: Echo Signal

After independent DBS and MIMO processing, the angles are interpolated on a common grid to form the MIMO-DBS image. Comparing (2) and (3), DBS provides finer resolution in the lateral directions, while MIMO performs best at the radar boresight. Thus, combined MIMO-DBS improves the resolution across the entire FoV of the radar.

**Figure 2:**
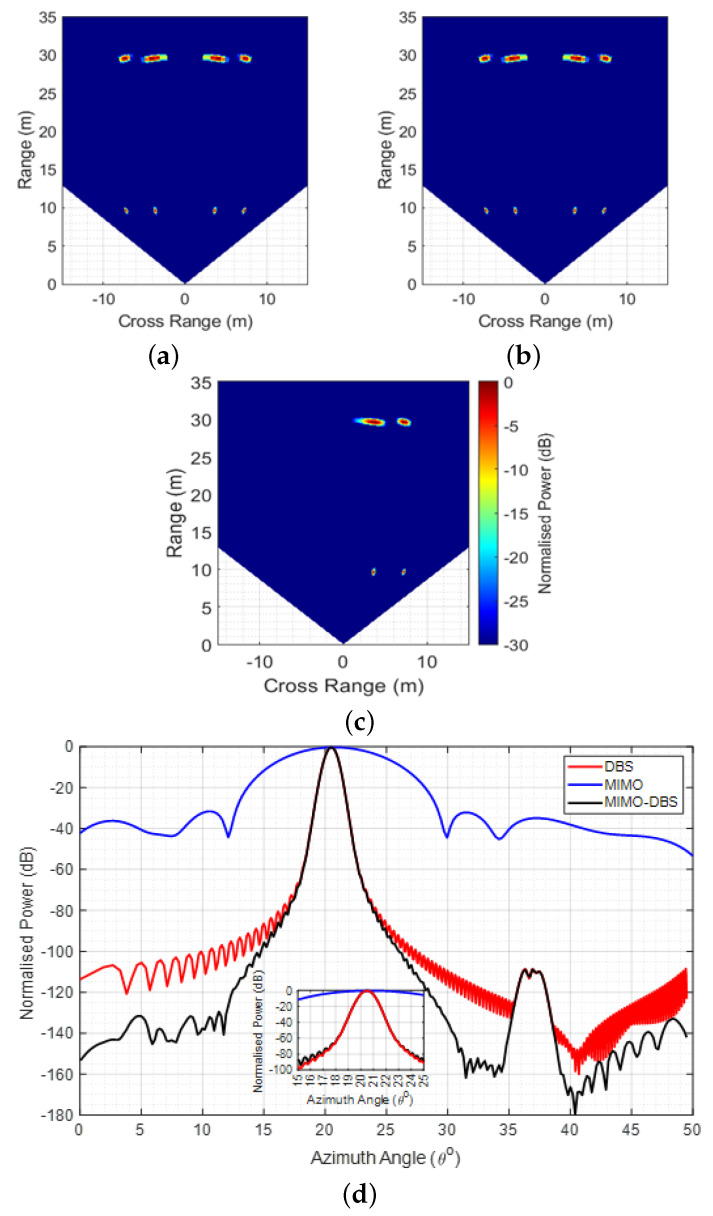
Modelled results at thermal noise background: (**a**) DBS; (**b**) MIMO; (**c**) MIMO−DBS; (**d**) range−cuts for the target at (3.6, 10) m.

For illustration, the modelled results for four point targets are shown in Figure 2. The target coordinates (cross-range, range) are (3.6, 10), (7.2, 10), (3.6, 30), and (7.2, 30) m, and the HR is at (0, 0) m. The scenario is modelled based on 77 GHz INRAS Radarbook with M=4 and N=8 and 1 GHz bandwidth. The HR moves at 40km/h and $T_{F}=64\;\mathrm{ms}$. To highlight the impact of each processing step on resolution, a 30 dB dynamic range is used. The left and right side ambiguity [26] due to the cosine relationship in (3) causes false targets at image angles after DBS, which are removed after MIMO-DBS processing, with an improvement in SLL. The latter is highlighted using range cuts at 10 m in Figure 2d.

### 2.2. Multi-Modal Processing for Interference

We now consider a case where, in addition to the echo signal, an interfering signal is also received at the HR (hereafter termed the victim radar (VR)). Interference is assumed to originate from an external radar (interfering radar, IR) and is therefore modelled with a one-way propagation delay. The phase of FMCW interference $\phi_{\mathrm{IF}-i}\left(t,t_{s}\right)$ at the IF stage is

(4) $$\phi_{\mathrm{IF}-i}\left(t,t_{s}\right)=2\pi \;\left(0.5\;\Delta K\;t^{2}+K_{i}\;t\;\tau_{i}\left(t_{s}\right)+\Delta F\;t\right.\left.-0.5\;K_{i}\;\tau_{i}^{2}\left(t_{s}\right)+F_{i}\;\tau_{i}\left(t_{s}\right)\right)$$

where $\Delta K=K_{v}-K_{i}$, $K_{i}$ is the IR sweep rate, $\Delta F=F_{v}-F_{i}$, and $F_{i}$ is the IR carrier frequency.

#### 2.2.1. DBS Processing

The instantaneous IF frequency of the interference is

(5) $$f_{\mathrm{IF}-i}\left(t,t_{s}\right)=\Delta K\;t+K_{i}\;\tau_{i}\left(t_{s}\right)+\Delta F$$

$f_{\mathrm{IF}}-i$ changes across slow-time due to different waveform parameters of VR and IR, introducing an additional phase shift $\phi_{\mathrm{int}}$ after range and Doppler compressions. For one-way propagation delay of interference, the Doppler frequency of interference, $f_{D-i}$, is

(6) $$f_{D-i}\left(t_{s}\right)=\frac{v_{p}}{\lambda }\cos\;\theta_{\mathrm{SB}-i}\left(t_{s}\right)+\frac{1}{2\pi }\;\frac{d\phi_{\mathrm{int}}\left(t_{s}\right)}{dt_{s}},$$

where $\theta_{\mathrm{SB}-i}\left(t_{s}\right)$ denotes the DBS sub-beam angle associated with interference. Depending on the parameters of VR and IR, the second term in (6) introduces a random phase shift. After mapping to the DBS angle, as in (2), the following is obtained.

(7) $$\theta_{\mathrm{SB}-i}\left(t_{s}\right)=\arccos\;\left[\frac{\lambda }{v_{p}}\left(f_{D-i}\left(t_{s}\right)-\frac{1}{2\pi }\;\frac{d\phi_{\mathrm{int}}\left(t_{s}\right)}{dt_{s}}\right)\right].$$

#### 2.2.2. Interference Classes Based on Doppler Characteristics

The waveform parameters of IR relative to VR define distinct characteristics of interference in the Doppler domain, broadly classified as synchronous, semi-synchronous, and asynchronous cases. The latter is more common and can be further categorised into:

- Periodic interference, which occurs at the same fast-time of all VR chirps in a CPI.
- Semi-periodic interference, which occurs at different fast-time for a subset of chirps, after which the sequence repeats and becomes periodic over the CPI.
- Aperiodic interference, which occurs at different fast-time across all VR chirps in a CPI.

**Figure 3:**
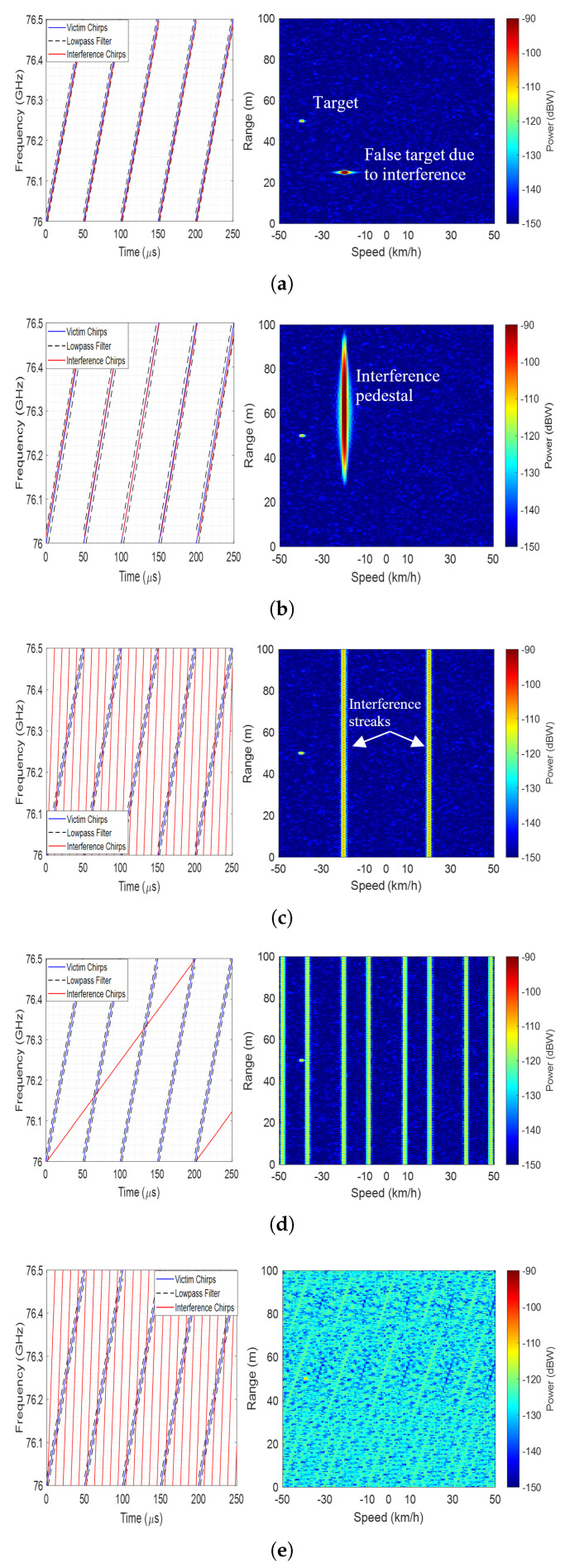
Classification of radar interference with chirp overlap and range−Doppler plots. (**a**) Synchronous; (**b**) semi−synchronous; (**c**) periodic (fast−chirp sequence); (**d**) semi−periodic; (**e**) aperiodic.

Example parameters for these interferences are listed in Table 1 for a VR with sweep time $T_{\mathrm{sv}}=50$ μs and bandwidth $B_{\mathrm{sv}}=$ 0.5 GHz. $T_{\mathrm{sint}}$ and $B_{\mathrm{sint}}$ denote sweep time and bandwidth of the IR. Modelled chirp overlays and corresponding range-Doppler maps are shown in Figure 3 for a representative case with a collocated target and interferer at 50 m, $10^{\circ }$ relative to the VR boresight, with the VR moving at 40 km/h.

As shown in Figure 3, each interference class exhibits distinct characteristics after range and Doppler compressions. Accordingly, the slow-time phase-rate term in (6) satisfies

(8) $$\frac{d\phi_{\mathrm{int}}\left(t_{s}\right)}{dt_{s}}=\left\{\begin{array}{cc} 0, & \mathrm{synchronous}\;\mathrm{and}\;\mathrm{semi-synchronous},\\ C\neq 0, & \mathrm{periodic}/\mathrm{semi-periodic},\\ A(t_{s}), & \mathrm{aperiodic}. \end{array}\right.$$

Here, *C* is a non-zero constant, and $A(t_{s})$ denotes a non-zero random value, both determined by the relative waveform parameters of VR and IR. Based on these characteristics,

1. For synchronous and semi-synchronous cases, interference is focused at a Doppler bin.
2. For periodic and semi-periodic cases, due to different $K_{v}$ and $K_{i}$, the second term in (6) is non-zero, introducing a ‘quasi-periodic’ phase shift. This leads to interference spread across a few Doppler bins. For periodic case, the number of Doppler bins affected by interference, $N_{\mathrm{DINT}}$, is equal to 2 in a real (single) channel receiver as interference folds over both positive and image side of spectrum [11]. $N_{\mathrm{DINT}}=1$ in a dual (in-phase/quadrature (I/Q)) channel receiver. In semi-periodic case, $N_{\mathrm{DINT}}$ is equal to the number of VR chirps over which interference sequence is aperiodic. In the discussed example, m = 4 (Table 1), leading to $N_{\mathrm{DINT}}=4$ in an I/Q channel receiver and $N_{\mathrm{DINT}}=8$ in a real-channel receiver.
3. For aperiodic case, interference energy spreads across the Doppler bins $\left(N_{\mathrm{DINT}}=N_{\mathrm{CPI}}\right)$.

Given the distinct appearance of interference, it is reasonable to expect that DBS processing will impact each case in a specific way, as discussed in the subsequent sections.

#### 2.2.3. MIMO Beamforming: Interference

The Rx elements of VR receive interference originating from an external transmitter with timing and waveform parameters generally not coherent with the VR virtual array. Consequently, after spatial compression, the beampattern associated with interference reception is defined by *N*-element physical Rx aperture of VR, causing a wider interference beam compared with the echo signal, for which the virtual aperture is coherently combined.

## 3. Signal Processing Gain

In this section, we analyse signal processing gain when a signal composed of echo returns and interference is processed at the VR. The processing chain is composed of analogue processing, implemented at the front end and digital signal processing (DSP).

### 3.1. Receiver Input

For free-space propagation, the received echo power (linear units) from a point target located at (θ,ϕ) (azimuth, elevation) is given by

(9) $$P_{R}\left(\theta ,\phi \right)=\frac{P_{\mathrm{Tx}}\;G_{\mathrm{Tx}}\left(\theta ,\phi \right)\;G_{\mathrm{Rx}}\left(\theta ,\phi \right)\;\lambda^{2}\;\sigma }{{\left(4\pi \right)}^{3}\;R_{T}^{4}\;L_{p}}$$

where $P_{\mathrm{Tx}}$ is the VR transmitted power; $G_{\mathrm{Tx}}\left(\theta ,\phi \right)$ and $G_{\mathrm{Rx}}\left(\theta ,\phi \right)$ are the VR transmit and receive antenna gains towards the target direction, respectively; σ is the target radar cross section (RCS); $R_{T}$ is range to target; and $L_{P}$ represents miscellaneous losses.

The interference received at VR is assumed to propagate one-way from an IR located at $\left(\theta_{\mathrm{Int}},\phi_{\mathrm{Int}}\right)$ with received power (linear units):

(10) $$P_{\mathrm{RInt}}\;\left(\theta_{\mathrm{Int}},\phi_{\mathrm{Int}}\right)=\frac{P_{\mathrm{TxI}}\;G_{\mathrm{TxI}}\;\left(\theta_{\mathrm{Int}},\phi_{\mathrm{Int}}\right)\;G_{\mathrm{Rx}}\;\left(\theta_{\mathrm{Int}},\phi_{\mathrm{Int}}\right)\;\lambda^{2}}{{\left(4\pi R_{I}\right)}^{2}\;L_{p}}$$

where $P_{\mathrm{TxI}}$ is the transmitted power of IR, $G_{\mathrm{TxI}}\left(\theta_{\mathrm{Int}},\phi_{\mathrm{Int}}\right)$ is the transmit antenna gain of IR transmitter towards the VR, $G_{\mathrm{Rx}}\left(\theta_{\mathrm{Int}},\phi_{\mathrm{Int}}\right)$ is the receive antenna gain of VR towards the IR, and $R_{I}$ is the range to the interferer.

The thermal noise power at receiver over an equivalent noise bandwidth *B* is $P_{N}=kT_{s}B$, with Boltzmann constant *k* and system noise temperature $T_{s}$. The input SINR is

(11) $${\mathrm{SINR}}_{\mathrm{RxIn}}=\frac{P_{R}\left(\theta ,\phi \right)}{P_{\mathrm{RInt}}\left(\theta_{\mathrm{Int}},\phi_{\mathrm{Int}}\right)+P_{N}}.$$

Due to one-way propagation of interference, at shorter ranges, interference power most likely dominates thermal noise floor [29]. Therefore, in the remainder of the discussion, we consider interference as the main contributor to the noise plus interference floor.

The received signal is downconverted and low-pass-filtered, which limits interference duration and results in the appearance of chirp-like interference pulses.

### 3.2. Analogue Front End (AFE)

The AFE provides gain and filtering prior to digitisation. For notational clarity, we express the net AFE gain in dB as

(12) $$G_{\mathrm{AFE}}=G_{\mathrm{LNA}}+G_{\mathrm{VGA}}-L_{\mathrm{Mixer}}-L_{\mathrm{FS}}+G_{\mathrm{ADC}}$$

where $G_{\mathrm{LNA}}$ and $G_{\mathrm{VGA}}$ are the gains of the low-noise amplifier and variable gain amplifier, respectively; $L_{\mathrm{Mixer}}=20\log0.5$ is the loss due to double-sideband to single-sideband conversion at the mixer; $L_{\mathrm{FS}}$ is the loss due to ADC saturation; $G_{\mathrm{ADC}}=6.02N_{\mathrm{ADC}}+1.74$ is the ADC gain [30]; and $N_{\mathrm{ADC}}$ is the number of ADC bits. INR at the output of AFE is

(13) $${\mathrm{INR}}_{\mathrm{AFE}}=\frac{P_{\mathrm{RInt}}\;\left(\theta_{\mathrm{Int}},\phi_{\mathrm{Int}}\right)\;G_{\mathrm{AFE}}^{\prime }}{P_{N}}$$

where $G_{\mathrm{AFE}}^{\prime }$ is the AFE gain in linear units. Both the echo signal and interference will experience similar processing gain at AFE. Consequently, the equivalent gain in SINR is close to 0.

### 3.3. Digital Signal Processing

#### 3.3.1. Range Compression

Range compression is performed by an $N_{\mathrm{RC}}$-point FFT across fast-time. After this, the usable frequency range is from 0 to $F_{\mathrm{Max}}$. $F_{\mathrm{Max}}$ in a single-channel receiver is $F_{s}/2$, where $F_{s}$ is the sampling frequency. Frequency bin resolution $\left(FFT_{\mathrm{BW}}\right)$ is defined by $F_{s}/N_{\mathrm{RC}}$, and the equivalent FFT noise bandwidth reduction gain is

(14) $$G_{\mathrm{FFT}}=\frac{F_{\max}}{{\mathrm{FFT}}_{\mathrm{BW}}}=\frac{F_{s}/2}{F_{s}/N_{\mathrm{RC}}}=\frac{N_{\mathrm{RC}}}{2}$$

The beat frequency of the echo signal is a constant frequency tone, occupying a single frequency bin after range compression (see Figure 3). In contrast, time-limited interference spreads over multiple frequency bins, defined by the fast-time dependent first term in (5). Hence, the product of the number of frequency bins with interference, $N_{\mathrm{INT}}$, and FFT resolution, $1/T_{\mathrm{sv}}$, should be $\Delta K\;\cdot \;T_{\mathrm{sv}}$. Therefore, $N_{\mathrm{INT}}=\Delta K\;T_{\mathrm{sv}}^{2}.$ For a larger value of $N_{\mathrm{INT}}$, interference spreads over a larger number of frequency bins with a lower mean power. The power of time-localised interference may further be reduced depending on its temporal position within the spectral window. We define this with an attenuation factor $W_{\mathrm{FastFFT}}^{*}$ [11].

Thermal noise spreads uniformly, and the equivalent noise power per frequency bin depends upon frequency resolution [31], which in a single-channel receiver is

(15) $$P_{N}=k\;T_{s}\left(\frac{N_{\mathrm{FFT}}}{2}\right)\;\frac{1}{T_{\mathrm{sv}}}$$

where $1/T_{\mathrm{sv}}$ is noise bandwidth per FFT bin, and $kT_{s}/T_{\mathrm{sv}}$ gives thermal noise floor level.

Based on the expressions derived above and the fact that the echo signal remains concentrated in a single range bin (for most of the cases), whereas interference energy is distributed over $N_{\mathrm{INT}}$ bins and further weighted by $W_{\mathrm{FastFFT}}^{*}$, SINR and INR after range compression, represented as ${\mathrm{SINR}}_{R}$ and ${\mathrm{INR}}_{R}$, respectively, are estimated as

(16) $$\begin{array}{cc} {\mathrm{SINR}}_{R}\; & ={\mathrm{SINR}}_{\mathrm{RxIn}}\;\frac{G_{\mathrm{FFT}}}{\;G_{\mathrm{FFT}}/\left(N_{\mathrm{INT}}\;W_{\mathrm{FastFFT}}^{*}\right)\;}={\mathrm{SINR}}_{\mathrm{RxIn}}\;N_{\mathrm{INT}}\;W_{\mathrm{FastFFT}}^{*},\\ {\mathrm{INR}}_{R}\; & ={\mathrm{INR}}_{\mathrm{AFE}}\;\frac{G_{\mathrm{FFT}}/\left(N_{\mathrm{INT}}W_{\mathrm{FastFFT}}^{*}\right)}{\;G_{\mathrm{FFT}}/T_{\mathrm{sv}}\;}={\mathrm{INR}}_{\mathrm{AFE}}\;\frac{T_{\mathrm{sv}}}{N_{\mathrm{INT}}\;W_{\mathrm{FastFFT}}^{*}}. \end{array}$$

In an I/Q channel receiver, an interference power reduction by a factor of 2 is expected.

#### 3.3.2. Range-Doppler Compressions

The received chirps are integrated over a CPI during Doppler compression, resulting in echo signal power increase by a factor of $N_{\mathrm{CPI}}^{2}$ for coherent chirps, while noise power increases by a factor of $N_{\mathrm{CPI}}$. As discussed earlier, for a majority of cases, interference is non-coherent across the VR chirps. Therefore, similar to noise, its power should increase by $N_{\mathrm{CPI}}$ times if all the victim chirps within a CPI are interfered with. However, due to different hardware configurations and waveform parameters of VR and IR, only a subset of VR chirps are interfered with [11], represented as $N_{\mathrm{CPI-INT}}$, with $N_{\mathrm{CPI-INT}}\leq N_{\mathrm{CPI}}$. This defines the probability of interference occurrence as $P_{\mathrm{INT-CPI}}=N_{\mathrm{CPI-INT}}/N_{\mathrm{CPI}}$. Based on the value of $P_{\mathrm{INT-CPI}}$, following cases can occur:

1. If $P_{\mathrm{INT-CPI}}=0$, none of the chirps within Doppler frame have interference (SINR = SNR).
2. If $P_{\mathrm{INT-CPI}}=1$, $N_{\mathrm{CPI-INT}}=N_{\mathrm{CPI}}$, and all chirps within a Doppler frame have interference, resulting in maximum interference power.

SINR and INR after range and Doppler compressions, represented as ${\mathrm{SIN}R}_{\mathrm{RD}}$ and ${\mathrm{INR}}_{\mathrm{RD}}$, respectively, for a real-only channel receiver, are estimated as follows:

(17) $$\begin{array}{cc} {\mathrm{SINR}}_{\mathrm{RD}}\; & ={\mathrm{SINR}}_{R}\;\frac{N_{\mathrm{CPI}}^{2}}{P_{\mathrm{INT-CPI}}\;\left(N_{\mathrm{CPI}}/2\right)}={\mathrm{SINR}}_{R}\;\frac{2\;N_{\mathrm{CPI}}}{P_{\mathrm{INT-CPI}}},\\ {\mathrm{INR}}_{\mathrm{RD}}\; & ={\mathrm{INR}}_{R}\;\frac{P_{\mathrm{INT-CPI}}\;\left(N_{\mathrm{CPI}}/2\right)}{N_{\mathrm{CPI}}}={\mathrm{INR}}_{R}\;\frac{P_{\mathrm{INT-CPI}}}{2}. \end{array}$$

A factor of 2 is included as interference impacts both positive and image-frequency bins.

#### 3.3.3. Spatial Compression

In time division multiplexing (TDM) MIMO, the Tx elements of VR transmit at different time slots, and after virtual array formation, the echo signal has a $\;{\left(M\;\cdot \;N\right)}^{2}$ times increase in power. In contrast, for asynchronous interference, coherence across *M* Tx time slots is not preserved. Furthermore, only a subset of received chirps at VR are affected by interference, defining the probability of interference occurrence as

(18) $$P_{\mathrm{Int-Az}}=\frac{N_{\mathrm{Int-MIMO}}}{M\;\cdot \;N}$$

where $N_{\mathrm{Int-MIMO}}$ represents the number of chirps within the azimuth frame with interference and $N_{\mathrm{Int-MIMO}}\leq M\;\cdot \;N$. Under these assumptions, SINR and INR after spatial compression, ${\mathrm{SIN}R}_{\mathrm{Az}}$ and ${\mathrm{INR}}_{\mathrm{Az}}$, respectively, are

(19) $$\begin{array}{cc} {\mathrm{SINR}}_{\mathrm{Az}}\; & ={\mathrm{SINR}}_{\mathrm{RD}}\;\frac{{\left(M\;\cdot \;N\right)}^{2}}{P_{\mathrm{Int-Az}}\;\;\cdot \;M\;\cdot \;N}={\mathrm{SINR}}_{\mathrm{RD}}\;\frac{M\;\cdot \;N}{P_{\mathrm{Int-Az}}},\\ {\mathrm{INR}}_{\mathrm{Az}}\; & ={\mathrm{INR}}_{\mathrm{RD}}\;\frac{P_{\mathrm{Int-Az}}\;\;\cdot \;M\;\;\cdot \;N}{M\;\;\cdot \;N}={\mathrm{INR}}_{\mathrm{RD}}\;P_{\mathrm{Int-Az}}. \end{array}$$

#### 3.3.4. MIMO-DBS Processing

For a common grid point (*x*, *y*), defined by (range, angle), that lies between four points at coordinates ($x_{1}$, $y_{1}$), ($x_{2}$, $y_{1}$), ($x_{1}$, $y_{2}$), ($x_{2}$, $y_{2}$) with intensities *f*($x_{1}$, $y_{1}$), *f*($x_{2}$, $y_{1}$), *f*($x_{1}$, $y_{2}$), *f*($x_{2}$, $y_{2}$), where *x* represents range, *y* represents angle, and $y_{1}$ and $y_{2}$ represent MIMO and DBS angles, respectively, we estimate MIMO and DBS angle intensity values at (*x*, *y*) using

(20) $$\begin{array}{cc} f_{\mathrm{MIMO}}\left(x,y\right)\; & =f\left(x_{1},y_{1}\right)\;\frac{x_{2}-x}{x_{2}-x_{1}}+f\left(x_{2},y_{1}\right)\;\frac{x-x_{1}}{x_{2}-x_{1}},\\ f_{\mathrm{DBS}}\left(x,y\right)\; & =f\left(x_{1},y_{2}\right)\;\frac{x_{2}-x}{x_{2}-x_{1}}+f\left(x_{2},y_{2}\right)\;\frac{x-x_{1}}{x_{2}-x_{1}}. \end{array}$$

Next, for $y_{1}=\theta_{\mathrm{t-MIMO}}$ and $y_{2}=\theta_{\mathrm{SB}-t_{s}}$, we perform an interpolation for MIMO-DBS using

(21) $$f_{\mathrm{MIMO-DBS}}\left(x,y\right)=f_{\mathrm{MIMO}}\;\frac{y_{2}-y}{y_{2}-y_{1}}+f_{\mathrm{DBS}}\;\frac{y-y_{1}}{y_{2}-y_{1}}$$

As discussed earlier, unlike the echo signal, interference appears at different MIMO and DBS angles (see (3) and (7)). Therefore, after interpolation, interference power is reduced if $f_{\mathrm{MIMO}}$ and $f_{\mathrm{DBS}}$ are different at grid point (*x*, *y*), quantified in the next section.

### 3.4. Multiple Interferers

For *Q* independent interference sources, the total received interference power at the receiver input can be expressed as

(22) $$P_{\mathrm{RInt}}=\sum_{q=1}^{Q}P_{\mathrm{RInt},q}.$$

After a given processing stage *s*, each interferer experiences a different weighting $\eta_{\mathrm{sq}}$, which depends on its waveform parameters, temporal overlap, and spreading behaviour in range, Doppler, and angle. Therefore, the mean interference power after stage *s* can be written as

(23) $$P_{I,s}=\sum_{q=1}^{Q}\eta_{\mathrm{sq}}\;P_{\mathrm{RInt},q},$$

Accordingly, the corresponding INR and SINR can be expressed as

(24) $${\mathrm{INR}}_{s}=\frac{P_{I,s}}{P_{N,s}},\;{\mathrm{SINR}}_{s}=\frac{P_{R,s}}{P_{I,s}+P_{N,s}},$$

where $P_{R,s}$ and $P_{N,s}$ represent the echo signal power and noise power after the *s*th processing stage, respectively.

In practice, interference signals from different sources are generally not coherent across the processing chain. Therefore, the equivalent interference floor is typically governed by the strongest interferer, while the remaining interferers primarily make the interference floor more uniform with Gaussian-like statistics as the number of independent sources increases [28].

## 4. Interference to Noise Ratio Heatmaps

In our previous work, we introduced a processing gain heatmap to predict the effect of a wide range of waveform parameters on signal-to-interference ratio (SIR) after range compression [29]. Here, we extend the analysis by presenting heatmaps after subsequent processing stages, specifically after Doppler and spatial compressions and MIMO-DBS processing, to establish application boundaries for MIMO-DBS. To quantify this, we evaluated INR as a function of the parameters $B_{\mathrm{sv}}/B_{\sin t}$ and $T_{\mathrm{sv}}/T_{\sin t}$, estimated using

(25) $$\mathrm{INR}=\frac{E\;\left[P_{I}\right]}{P_{N}}$$

where $P_{N}$ is the thermal noise floor of VR and $E[P_{I}]$ is the mean interference power calculated across range bins where the target is spatially located.

**Figure 4:**
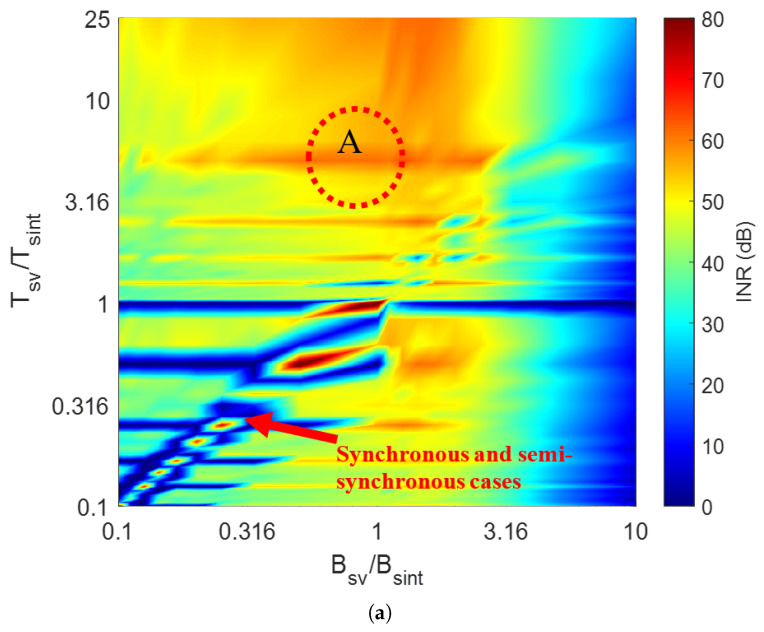

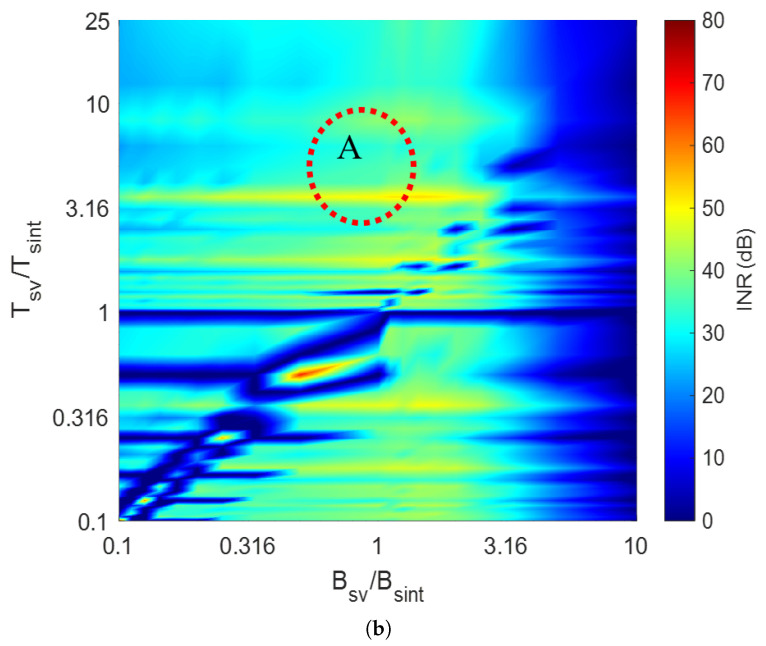
Interference to noise ratio (INR) heatmaps as a function of VR and IR waveform parameters. (**a**) MIMO, (**b**) MIMO-DBS. Encircled region A represents an example of periodic interference.

To illustrate the use of INR heatmaps, we present a use case where VR is moving at 40km/h, configured according to Inras Radarbook chipset, with *T*_sv_ = 50 μs, $B_{\mathrm{sv}}=0.5\;\mathrm{GHz}$, and $T_{F}=10\;\mathrm{ms}$. The IR waveform parameters are varied to generate different interference classes. The source of interference is at 20 m from VR along its boresight, and a point target is at 50 m from VR, also along its boresight. The corresponding heatmaps after MIMO and MIMO-DBS processing are presented in Figure 4a and Figure 4b, respectively, showing a significant reduction in INR after MIMO-DBS for most VR and IR parameter relationships.

### 4.1. Synchronous and Semi-Synchronous Interference

**Figure 5:**
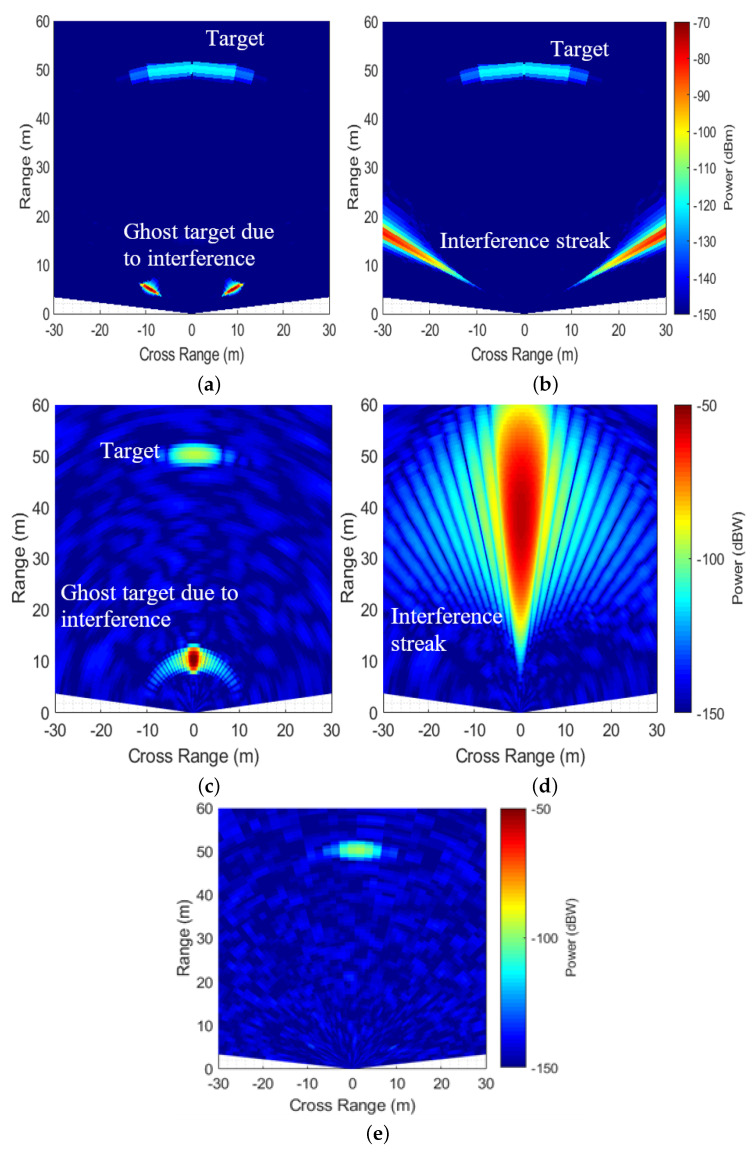
Range-cross range plots. (**a**) DBS: synchronous interference; (**b**) DBS: semi-synchronous interference; (**c**) MIMO: synchronous interference; (**d**) MIMO: semi-synchronous interference; (**e**) MIMO-DBS: synchronous and semi-synchronous interference.

Synchronous and semi-synchronous interference are the most critical types of interference as they appear as focused ghost targets after signal processing. In Figure 4, such cases appear along the diagonal region, and MIMO-DBS processing significantly improves their INR. The DBS-only results of synchronous and semi-synchronous interference $\left(B_{\mathrm{sv}}/B_{\mathrm{sint}}=1.007,T_{\mathrm{sv}}/T_{\mathrm{sint}}=1\right)$ are shown in Figure 5a and Figure 5b, respectively. Due to the cosine relationship in (3), only one side of the azimuth axis presents an unambiguous FoV. Although both target and interferer are located at the VR boresight, they are mapped to different DBS angles, hence separated along the angular dimension. Furthermore, as DBS resolution improves in the lateral directions, the echo signal at the boresight has coarser resolution compared to the interference streak at ≈70°. Consequently, a smaller number of angle bins are affected by interference.

MIMO beamformed images for these cases are presented in Figure 5c and Figure 5d, respectively. A “ghost target” of high power appears for synchronous interference, while semi-synchronous interference produces an interference streak, masking the target. Notably, in both cases, the echo signal and interference appear at VR boresight in accordance with their true AoAs. After MIMO-DBS beamforming, owing to different MIMO and DBS angles of interference, interpolation according to (21) suppresses interference power for both cases (Figure 5e), while the target being at the same MIMO and DBS angle is focused, resulting in an SINR improvement of 60 dB in this case.

### 4.2. Asynchronous Interference

#### 4.2.1. Periodic and Semi-Periodic Interference

**Figure 6:**
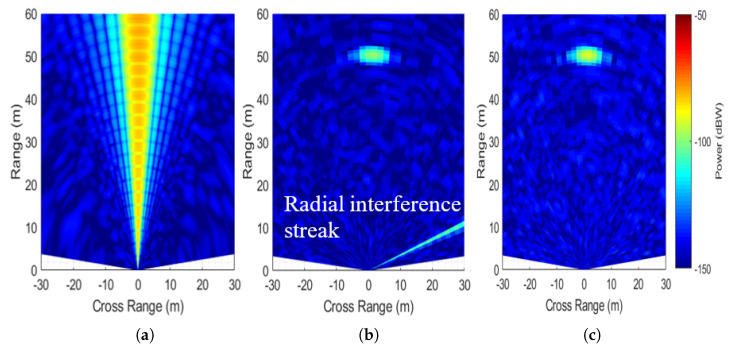
Range-cross range plots for periodic interference with $B_{\mathrm{sv}}/B_{\mathrm{sint}}=1,T_{\mathrm{sv}}/T_{\mathrm{sint}}=5$. (**a**) MIMO. (**b**) MIMO−DBS: real−channel receiver. (**c**) MIMO−DBS: dual−channel receiver.

A MIMO beamformed image at the background of periodic interference with $B_{sv}/B_{sint}=1$, $T_{sv}/T_{sint}=5$ (region A in Figure 4a is shown in Figure 6a). Here, the target is fully masked by the interference beam. MIMO-DBS images for real and I/Q channel receivers are shown in Figure 6b and Figure 6c, respectively. For a real-channel receiver, a radial interference streak appears due to the image Doppler frequency. As the second phase term in (6) is constant, an interference streak is focused with finer resolution but well-separated from the echo signal. The use of an I/Q channel receiver eliminates interference. Semi-periodic interference affects a larger number of Doppler bins (Figure 3). Hence, the number of interference streaks after MIMO-DBS increases but with a reduced mean power.

#### 4.2.2. Aperiodic Interference

**Figure 7:**
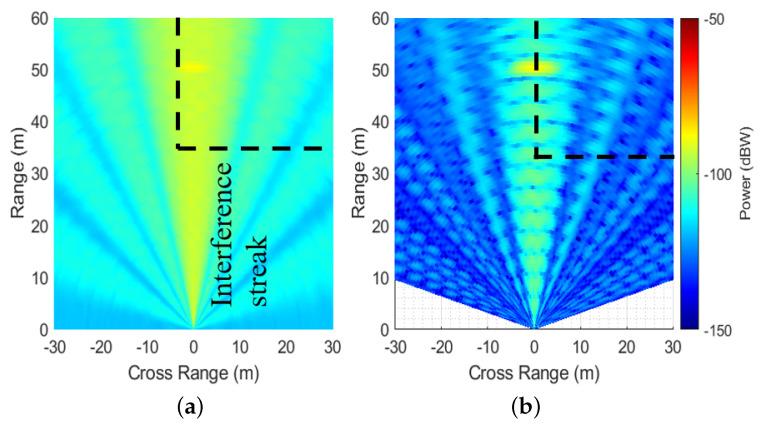
Range-cross range images for the aperiodic interference with $B_{sv}/B_{sint}=1,T_{sv}/T_{sint}=2.23$. (**a**) MIMO; (**b**) MIMO-DBS.

This corresponds to an area outside the diagonal region in Figure 4. MIMO and MIMO-DBS images for an example case with $B_{\mathrm{sv}}/B_{\mathrm{sint}}=1,T_{\mathrm{sv}}/T_{\mathrm{sint}}=2.23$ are shown in Figure 7a and Figure 7b, respectively. In this case, SINR improvement of ≈10 dB is observed after MIMO-DBS if the interference power is averaged along boresight (where DBS is not efficient). By averaging the interference power within the upper right quarter area (indicated by the black dashed box in Figure 7), SINR improvement of ≈38 dB is observed.

### 4.3. Performance Analysis for Multiple Interferers

To assess the robustness of MIMO-DBS in multiple interferer cases, we evaluated INR values for up to five interference sources, representing all types of interference classes with parameters stated in Table 2. The INR values were calculated after: MIMO, MIMO with time-domain interference mitigation using interference zeroing [19], MIMO-DBS, and MIMO-DBS with interference zeroing. We perform interference zeroing by detecting interference samples and replacing them with zeros. The results are detailed in Table 3, where Int1–Int5 are the interferer types/parameters obtained from Table 2.

MIMO-DBS achieves up to 35 dB greater interference suppression compared to the MIMO case. When combined with interference zeroing, suppression increases up to 60 dB with the interference floor reduced to the thermal noise floor level. Therefore, the proposed multi-modal technique, especially when augmented with simple-to-implement mitigation (interference zeroing), is highly effective in suppressing interference in complex environments.

**Figure 8:**
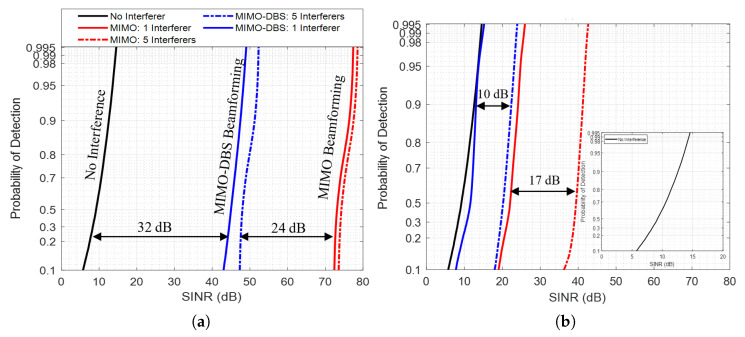
Probability of detection as a function of SINR. (**a**) Without interference zeroing, (**b**) with interference zeroing. Insert shows zoomed detection curve for signal detection against thermal noise.

Interference suppression is a critical requirement to optimise radar performance; however, it is essential to consider the associated trade-offs. For example, interference zeroing, while effective in reducing the interference floor, also removes a portion of the desired echo signal, which can reduce the coherent integration gain. This issue is particularly prominent when interference spans a larger number of fast-time samples or when multiple interferers are present. To quantify this trade-off, we evaluated detection performance for scenarios in Table 3 using a non-fluctuating (Swerling-0) target model and a probability of false alarm $P_{\mathrm{FA}}=10^{-6}$. The resulting detection curves are shown in Figure 8a and Figure 8b for the cases without and with zeroing, respectively.

The results show that MIMO-DBS reduces the interference floor to the level of the thermal noise floor when combined with interference zeroing. However, as the number of interferers increases, a large portion of fast-time samples are essentially zeroed out, which consequently degrades the detection performance, as shown in Figure 8b.

### 4.4. Effect of Waveform Modulation

The discussion presented in this paper is focused on the FMCW victim and interfering radars. Nevertheless, the MIMO-DBS interference suppression mechanism is more general in the sense that it depends on the mismatch between the spatial localisation of the echo and interference signals after MIMO and DBS processing. Therefore, similar suppression behaviour may also be expected for non-FMCW interference sources, such as phase-modulated continuous wave or pulse-Doppler radars, provided that the interference remains decorrelated from the desired echo across the processing chain.

## 5. Experimental Setup and Analysis

To evaluate the impact of MIMO-DBS, experimental trials were conducted in automotive and maritime conditions. The results shown here are representative examples selected from multiple measurement runs conducted in the respective trial campaigns.

### 5.1. Automotive Measurements

**Figure 9:**
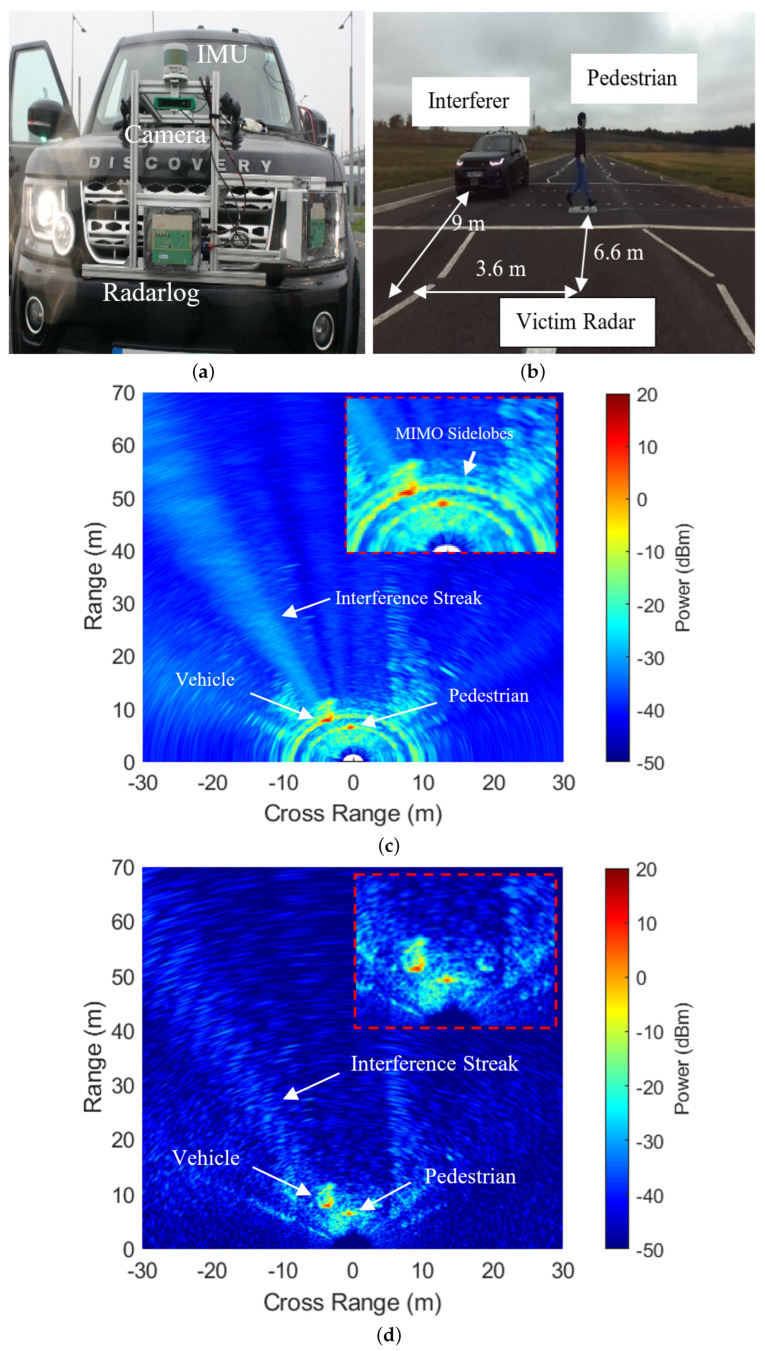
Measured results. (**a**) Host vehicle with VR. (**b**) Test setup. (**c**) MIMO beamforming, (**d**) MIMO-DBS beamforming. Inserts show the zoomed regions, highlighting the targets.

The dataset was collected at the test proving grounds of HORIBA MIRA [32] using time-stamped measurements from radar, lidar, stereo camera, and an inertial measurement unit (IMU). The 77 GHz radars, including INRAS Radarlog/Radarbook, operating in TDM-MIMO mode as VRs, were installed in front-looking/corner-looking orientations on a host vehicle (Figure 9a). Texas Instruments (TI) AWR1243 and NXP Dolphin transmitted from a single Tx element as IRs and were installed in front-looking/corner-looking orientations on target vehicle [33]. This configuration produces predominantly aperiodic interference. The parameters are given in Table 4.

The experimental setup is shown in Figure 9b. Both vehicles were moving towards each other in adjacent lanes while a pedestrian mannequin crossed in front of the VR. For the frame shown here, the interfering vehicle was at 9 m from VR, while the pedestrian was at the VR boresight at ≈6.6 m. The relative speed between VR and IR was 2 m/s. MIMO and MIMO-DBS beamformed images from front-looking VR are shown in Figure 9c and Figure 9d, respectively. As expected for aperiodic interference, a distinct interference streak appears in the direction of IR after MIMO beamforming. DBS does not provide resolution refinement for a pedestrian mannequin close to the boresight. However, regardless, MIMO-DBS shows a noticeable suppression of interference (12 dB), with a side lobe reduction.

### 5.2. Maritime Measurements

**Figure 10:**
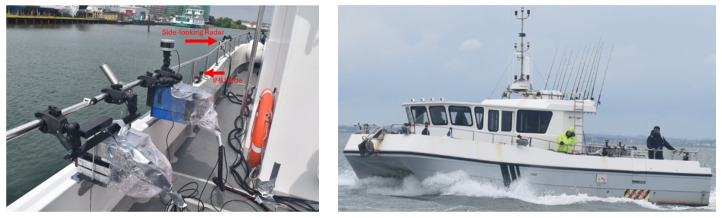
Measurement setup onboard the Valkyrie-6.

Measurements were conducted in the Solent, UK, in open sea and harbour water conditions, off the Isle of Wight, at sea state 2 under rainy and windy conditions. The sensing suite was installed on a 36-foot catamaran, which sailed from the Gosport Marina, UK. The sensor suite setup is shown in Figure 10 and comprises: (i) a 77 GHz Inras Radarlog with a 61-element virtual array, used in front and side-looking orientation relative to platform bearing; (ii) ZED cameras to provide time-stamped ground truth; and (iii) a xSens 680G IMU to record platform kinematics. Configuration parameters and expected performance estimates are given in Table 5.

**Figure 11:**
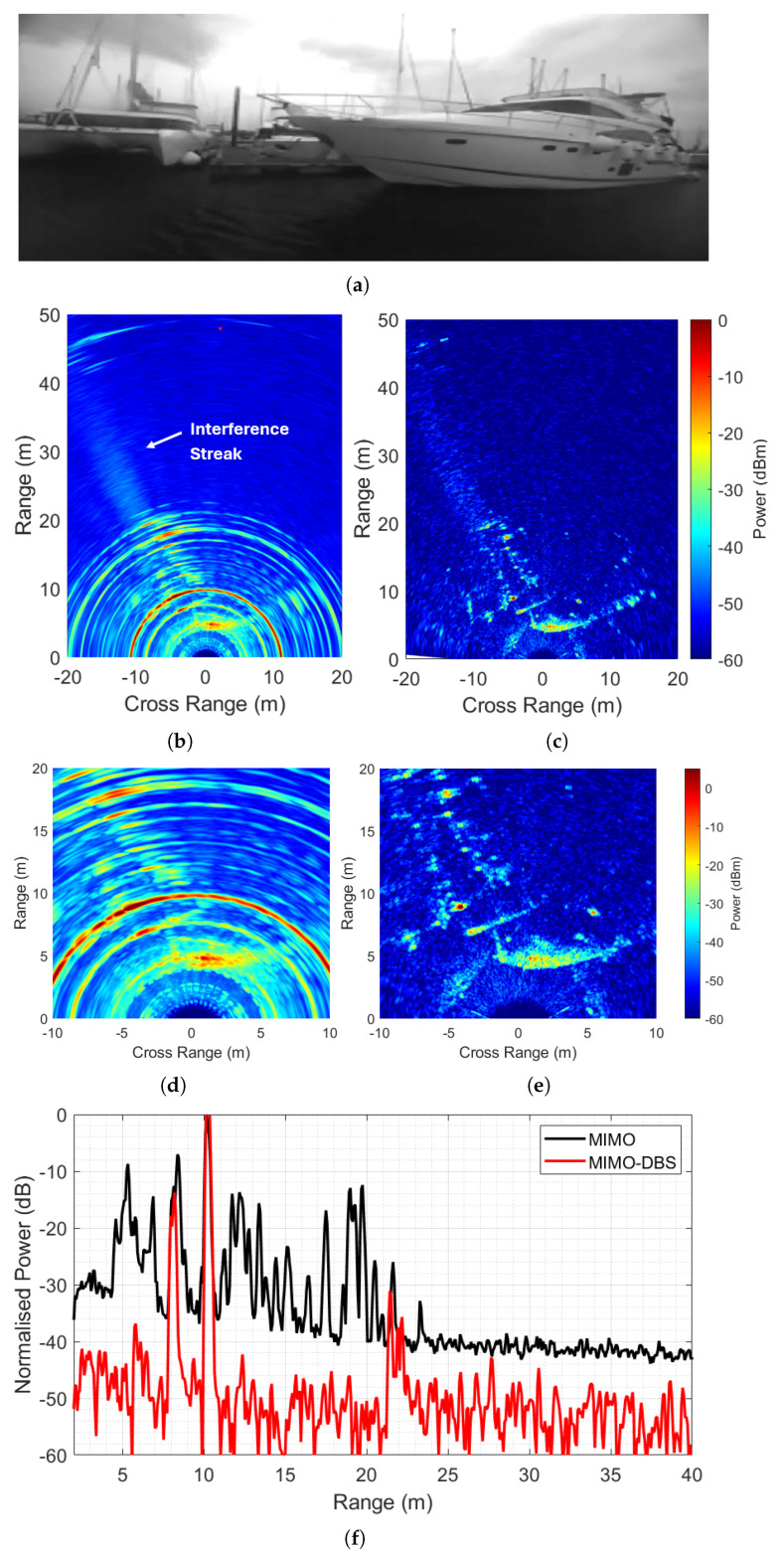
Measured results in maritime environment. (**a**) Ground truth of the harbour region. (**b**) MIMO beamformed image. (**c**) MIMO-DBS beamformed image. (**d**) Zoomed MIMO. (**e**) Zoomed MIMO-DBS. (**f**) Angle cut at −25 degrees, focusing on the interference streak.

Figure 11 shows the processed results after MIMO and MIMO-DBS beamforming from the perspective of the side-looking radar. The ground truth showing a docked boat next to the moving catamaran is shown in Figure 11a. Figure 11b,c present the MIMO and MIMO-DBS beamformed results, with an interference streak at −25 degrees. Here, side-looking radar receives interference from the signal of front-looking radars that gets reflected off the docked boat. Figure 11d,e show a zoomed region highlighting the resolution improvement and sidelobe suppression after MIMO-DBS, which not only shows docked boats but also the passable region for the platform boat, hence serving as an essential input to the path planning models. Figure 11f presents normalised angle cuts at −25 degrees showing a mean interference suppression of 12.5 dB in the presented case.

Maritime environments are typically sparse in nature, such as in the open-sea conditions. In such environments, the impact of mutual interference will not be too critical. However, in the dense conditions surrounding the infrastructure, harbour regions, the probability of interference, especially the self-interference, increases and hence must be addressed for reliable detection and tracking of marine objects.

### 5.3. Performance Analysis in a Dynamic Scenario

For dynamic scenarios, where both the VR and the targets are moving, adaptive MIMO-DBS processing has been discussed in [1], where the moving target is focused by tuning the relative velocity vector. Importantly, the interference suppression mechanism discussed in this work is preserved because interference still follows a different mapping in the MIMO and DBS beamformers compared to the echo signal. Moreover, increased relative motion reduces the coherence of interference across slow time, which further reduces the probability of synchronous, semi-synchronous, periodic, or semi-periodic interference.

### 5.4. Limitations of MIMO-DBS for Interference Suppression

MIMO-DBS is a signal processing technique, originally developed to enhance radar resolution, and adapted in this work to suppress interference. Its main strength is that it does not require waveform redesign or specialised hardware modifications and instead exploits the angular decorrelation of interference across the MIMO and DBS beamformers. This makes it particularly attractive for SoA radar platforms, where implementation simplicity and compatibility with existing processing chains are important.

The effectiveness of MIMO-DBS for interference suppression can be significantly reduced when the interference and the desired echo are mapped to the same angular bin after both MIMO and DBS beamforming. Such situations may arise in certain synchronous, periodic, or semi-periodic interference cases, particularly when the AoA of the desired target overlaps with that of interference originating from another source. Nevertheless, in the majority of operational scenarios, interference remains incoherent across the VR chirps, and therefore, a suppression effect after MIMO-DBS processing is still observed.

## 6. Conclusions

This paper presents a multi-modal interference suppression framework that exploits Doppler-domain disparity between the echo signal and the interference. By combining independent angle estimates from MIMO and DBS processes, the combined MIMO-DBS suppresses interference while preserving the coherent echo signal. The effectiveness of the approach has been demonstrated through extensive simulations spanning a broad range of VR and IR waveform relationships and interference classes, supported by the measured results from automotive and maritime environments. The results show that MIMO-DBS can provide substantial INR reduction, including multi-interferer cases, and can be further augmented by simple time-domain interference mitigation.

Although the proposed processing significantly improves SINR in many practical scenarios, particularly where interference pulses are uncorrelated and occur intermittently, dedicated interference mitigation remains necessary in situations requiring very high dynamic range, for example, when strong interference coexists with weak targets or when multiple targets exhibit large RCS disparities.

From an implementation perspective, DBS processing can be executed on millisecond time scales consistent with typical millimetre wave radars’ frame durations, and it does not require hardware modifications or substantial additional computational resources, making the approach attractive for deployment on SoA radar platforms. This is critical for path planning, safe manoeuvring, and docking of autonomous platforms, where accurate perception is essential within short time frames.

Future work will focus on extending MIMO-DBS to distributed and multi-sensor radar networks and evaluating interference suppression performance under networked operation and coordinated multi-platform autonomy conditions.

## Figures and Tables

**Table 1:** Types of interference and their conditions.

Interference Type	Condition and Parameters	
Synchronous	$K_{v}=K_{i};\;T_{\mathrm{sint}}=50$ μs, *B*_sint_ = 0.5 GHz	
Semi-Synchronous	$K_{v}\approx K_{i};\;T_{\mathrm{sint}}=50$ μs, *B*_sint_ = 0.495 GHz	
Asynchronous $K_{v}\neq K_{i}$	Periodic (Fast-chirp sequence)	Periodic (Slow-chirp sequence)
	$mT_{\mathrm{sint}}=T_{\mathrm{sv}},\;m=2,3,\ldots$	$T_{\mathrm{sint}}=mT_{\mathrm{sv}},\;m=2,3,\ldots$
	$T_{\mathrm{sint}}=10$ μs, *B*_sint_ = 0.5 GHz	
	Semi-Periodic	
	$mT_{\mathrm{sint}}=nT_{\mathrm{sv}},\;m=1,2,3,\ldots ,\;n=2,3,\ldots$	
	$T_{\mathrm{sint}}=200$ μs, *B*_sint_ = 0.5 GHz	
	Aperiodic	
	$mT_{\mathrm{sint}}\neq nT_{\mathrm{sv}},\;\mathrm{for}\;\mathrm{any}\;\mathrm{integers}\;m,n$	
	$T_{\mathrm{sint}}=10.3$ μs, *B*_sint_ = 0.5 GHz	

**Table 2:** Interferer’s parameters for multiple interference sources.

Interferer No./Type	Sweep Time (μs)	Bandwidth (GHz)	Range, Angle
Int1/Continuous wave	*∞*	0.0	20 m, $40^{\circ }$
Int2/Fast-chirp periodic	10	0.5	25 m, $30^{\circ }$
Int3/Aperiodic	41.3	0.5	30 m, $15^{\circ }$
Int4/Slow-chirp periodic	200	0.5	35 m, $7^{\circ }$
Int5/Semi-periodic	800	0.2	40 m, $2^{\circ }$

**Table 3:** Performance comparison for MIMO and MIMO-DBS combined with interference zeroing.

No. of Interferers	1 (Int1)	3 (Int1–Int3)	5 (Int1–Int5)
	INR (dB)		
MIMO Beamforming	62	65	66
MIMO Beamforming + Zeroing	12	20	28
MIMO-DBS Beamforming	37	34	31
MIMO-DBS Beamforming + Zeroing	1	8	11

**Table 4:** Radar configuration parameters for automotive trials.

Parameter	Radarlog	TI	NXP
Sweep Time (µs) (Tsv)	204.8	250	102.4
Bandwidth (MHz)	1000	950	1000
Start Frequency (GHz)	76	76.05	76.1
Pulse Repetition Interval (µs)	230	266	150
Antenna Configuration	4 × 16	1 × 4	1 × 4
Gain at Boresight (dBi)	14.4	10	17

**Table 5:** Configuration parameters for maritime trials.

Parameter	77 GHz Radar
Centre frequency (GHz)	77
Bandwidth (GHz)	2
Operational configuration	TDM MIMO
Array configuration (Tx × Rx)	4 × 16 (virtual 61 el)
Chirps per frame	4
Range resolution	0.075 m
Azimuth resolution	1.9° (virtual)
Coherent processing interval	128 ms
Platform speed	2.2 m/s
Unambiguous velocity	±0.95 m/s
Velocity resolution	0.015 m/s

## Data Availability

The data is available at: (i) [34] (ii) [35].
